# Intermittent preventive therapy for malaria: arguments in favour of artesunate and sulphamethoxypyrazine - pyrimethamine combination

**DOI:** 10.1186/1475-2875-10-70

**Published:** 2011-03-29

**Authors:** Frans Herwig Jansen

**Affiliations:** 1 ACT-ion Afrique, Brussels, Belgium

## Abstract

Recent publications put a serious warning regarding the inefficacy of sulphadoxine-pyrimethamine (SP) for the intermittent preventive treatment of malaria in young children (IPTi). Recommendations for other therapies are being made. By using a different and better sulphonamide (sulphamethoxypyrazine), it is possible to manufacture fixed dose combination pills with artesunate and pyrimethamine. This combination permits a full therapy over 24 hours (dosing interval being 12 hours). It is recommended that this combination should be tested in future field studies of IPTi.

## Background

Intermitted preventive therapy (IPT) is the administration of a full course of an anti-malarial drug at specified time points in pregnant women and children, whether parasites are present or not. It is expected that, by doing so, the incidence of clinical malaria will be reduced in the two patient groups. In pregnant women, the outcomes are the neonatal weight and the presence of placental malaria and maternal anemia. The idea behind the intermittent use of an anti-malarial drug is to lower the risks for spreading resistance and prevent the impairment of the development of natural immunity in children [1]. Currently the intermittent preventive treatment for malaria in infants (IPTi), children (IPTc) and pregnant women (IPTp) is performed with a sulphonamide (sulphadoxine) in combination with pyrimethamine, a combination which is no longer recommended for malaria treatment [2]. The use for IPTi is currently controversial as inconsistent data has been collected in different efficacy studies regarding the degree of protection against malaria, the period of protective benefit and the real positive effect on anaemia, hospital admission and mortality. The lower efficacy due to growing resistance to sulphadoxine-pyrimethamine and the high incidence of serious adverse effects in treated children are also issues of concern [3,4]. For instance, in a recent paper of Gosling *et al *comparing efficacy of SP, chlorproguanil-dapsone and mefloquine for preventing clinical malaria in infant, the first two regimens had no protective effect at all, and mefloquine had a protective effect of 38.1% [5]. Therefore, new drugs or drug combinations are needed for IPTi. Cairns *et al *suggested that "The ideal regimen for IPTi and IPT in older children would be a fixed formulation of two long-acting anti-malarials with similar pharmacokinetic profiles that could be given as a single dose and be perfectly safe" [6]. In a recent editorial, McGready suggested that preventive intermittent therapy in children below one year of age is probably not worth it since children are relatively protected from malaria in the first few months of their life [7]. She concluded that for IPTi, SP is the wrong drug in the wrong dose at the wrong time. This opinion contrasts with a meta-analysis involving six studies, published by Aponte *et al *where it was concluded that intermittent treatment with SP prevented malaria for about one month after each dose [8]. The point of view of McGready is interesting: following the loss of SP as a treatment regimen for symptomatic malaria, the question is whether the days of SP and similar drugs as preventive monotherapy are over? Probably yes. The arguments to conclude this will be developed in the following paragraphs.

## Sulphonamides for malaria

The use of sulphonamide in combination with pyrimethamine for the treatment of malaria goes back to the early seventies when Hoffman-La-Roche introduced the drug Fansidar^®^. It was recommended to use this combination as follows: three tablets containing 500 mg sulphadoxine and 25 mg pyrimethamine, as a single dose. Nearly at the same time a competitive drug was launched by Farmitalia under the brand name Metakelfin^® ^containing as sulphonamide sulphamethoxypyrazine (SM), but it was introduced in a limited number of countries only. Both SP and sulphamethoxypyrazine-pyrimethamine (SMP) have been used in Africa for many years and were only recently stopped being recommended for the curative treatment of malaria in some African countries in favour of artemisinin-based combination therapy (ACT). In the past, the combination with SP or SMP worked well, was cheap and well-accepted in virtually all areas where malaria is abundant.

The combination SP was selected by some for intermittent therapy in infant, children and in pregnant women. But why SP was preferred over SMP is not clear. Although both sulphonamides are relatively safe, it is known that fewer side effects have been seen with sulphamethoxypyrazine than with sulphadoxine [2]. In fact, SMP is still considered by many as the "best sulphonamide-pyrimethamine" combination. In fact, long ago the drug was strongly recommended by WHO and used in a large malaria eradication programme in the hyperendemic malarial area of Garki, Nigeria [9], and not a single side effect was reported in clinical efficacy trials.

SP and SMP act by inhibiting the folic acid biosynthesis of the parasite (but also of bacteria) at two levels. First, the sulphonamide acts as a competitive inhibitor with p-amino benzoic acid in the first part of the synthesis of folic acid. At a later stage, pyrimethamine interferes with the dihydrofolate reductase enzyme necessary to reduce the intermediate compound dihydrofolate into folic acid [10]. Unfortunately, parasites, including *Plasmodium falciparum*, can mutate around its action and SP resistance can be very high in certain areas like Ghana and Rwanda, where 35 and 50% resistance are reported, respectively [11,12]. Mutations can be manifold and are particularly directed against the reductase enzyme that is the target for pyrimethamine. Mutations against the synthase enzyme are more unlikely since the sulphonamide does not interfere with the enzyme system involved. From a practical point of view, this means that SP can become useless in case they are used in children suffering from an infection caused by resistant parasites, as it was probably the case in the patients studied by Gosling *et al *[5].

The use of combination therapies including artesunate or artemether could also be theoretically possible for IPT and represent a valid alternative to sulfonamide alone. Cissé *et al *published in 2006 their positive experience when using a single dose of artesunate combined with one dose of SP leading to a clear-cut prevention of malaria over a 13-week follow-up period [13]. However, using only one tablet of artesunate and one dose of SP is dangerous and may lead to the development of resistance since this combination is not capable of appropriately reducing the parasite load. If one would rather give a full treatment, making fixed dose preparations of artesunate with SP is not feasible since artesunate should be given in three doses over three days (48 h) and the SP part is administered as a single high dose (1,500 mg).

Interestingly, a single dose of 200 mg SMP gives a comparable therapeutic effect as 1,500 mg of SP. In fact despite the fact that both sulphonamides are considered to be long acting, SP has a longer elimination half life (about 200 h but variable) and a very high binding to plasma proteins (between 95 and 99%). In contrast the elimination half life of SMP is 65-85 hours and plasma protein binding is only 65% [14]. Since for sulphonamide therapy only the non-protein-bound part of the drug (the so called free fraction which distributes in all body compartments) is active against bacteria and parasites, a much smaller dose of SMP can be given when compared to SP. This also means that administering a larger dose of SMP (like the dose given for SP) results in a much longer duration of action than that of a comparable dose of SP.

Conversely to SP, it is possible to make a true fixed dose preparation of SMP and artesunate. This was done by the Belgian company Dafra Pharma which introduced this as a malaria therapeutic agent under the brand name Co-arinate^®^. Each pill for adults contains 200 mg artesunate, 500 mg sulphamethoxypyrazine and 25 mg pyrimethamine. The tablets for children contain half that dose and for infants the tablets can easily be broken so that the half unit contains only 50 mg artesunate with 125 mg sulphonamide and 6.25 mg pyrimethamine. Several studies involving several thousands of patients support the clinical efficacy against that combination [12,15-18]. The incidence of side effects is very low and adequate clinical and parasitological cure rates (APCR) PCR corrected at day 28 reaches up to 99% level. Interestingly, the drug is also highly efficacious in areas with pronounced SP resistance like in Rwanda [9]. The full curative dosing can be done over 24 h (dose interval 12h) or 48 h (dose interval 24 h).

In the author's opinion, the use of a FDC with artesunate should seriously be reconsidered for IPTi, and the combination As+SMP represents a good candidate drug for such use. In fact, the drug is highly efficacious, it is safe, simple to administer and its production cost is very low.

## Competing interests

The author declares that he has no competing interests. However, previously he was medical director of Dafra Pharma. He now retired from that function having reached the age.
